# Intracranial Carotid Occlusions: ADAPT versus SAVE and the role of Balloon Guide Catheters

**DOI:** 10.1007/s00062-023-01286-y

**Published:** 2023-04-26

**Authors:** Guglielmo Pero, Hugo Mota Dória, Mariangela Piano, Antonio Macera, Luca Quilici, Amedeo Cervo

**Affiliations:** 1 Department of Neuroradiology, Ospedale Niguarda Ca’ Granda, Piazza Ospedale Maggiore 3, 20162 Milano, Italy; 2 Department of Neuroradiology, Hospital Central do Funchal, Funchal, Madeira, Portugal; 3 Universidade da Madeira, Funchal, Madeira, Portugal; 4 Department of Neuroradiology, ASST Papa Giovanni XXIII, Bergamo, Italy

**Keywords:** Neurointervention, Stroke, Thrombectomy, Aspiration, Stentriever, Balloon guide catheter

## Abstract

**Purpose:**

Specific decisions made by neurointerventionists are often lost behind the data of large-scale trials, and many of these studies have taken place before the development of new techniques and devices. This study compares the stent-retriever assisted vacuum-locked extraction (SAVE) technique with a direct aspiration first pass (ADAPT), as well as the use of a balloon guide catheter (BGC), in intracranial internal carotid artery (IC-ICA) occlusions.

**Methods:**

Observational and retrospective study from an Italian hospital, including patients who underwent thrombectomy for IC-ICA occlusion between 1 January 2019 and 31 March 2021.

**Results:**

Out of 91 IC-ICA occlusions, the ADAPT was the first choice in 20 (22%) and the SAVE in 71 (78%). A BGC was used in 32 (35%) cases, always in conjunction with the SAVE technique. The use of SAVE technique without BGC was associated with the least risk of distal embolization (DE) in the territory occluded (44% vs. 75% when ADAPT technique was used; *p* = 0.03) and achieved first pass effect (FPE) more frequently (51% vs. 25%, *p* = 0.09). When the SAVE technique was used, BGC (BGC-SAVE) compared to no BGC (NoBGC-SAVE) was associated with a tendency for less DE (31% vs. 44%, *p* = 0.3), more FPE (63% vs. 51%, *p* = 0.5), the same median number of passes (1, *p* = 0.8) and similar groin-to-recanalization times (36.5 vs. 35.5 min, *p* = 0.5), none of which reached statistical significance.

**Conclusion:**

Our findings support the use of SAVE technique for IC-ICA occlusions; the added benefit of BGC compared to long sheaths was not remarkable in this sample.

## Introduction

Decisions regarding technical aspects of thrombectomy vary greatly in clinical practice [1]. The choices are often tailored by personal preferences with respect to a wide range of factors involving the specific case at hand; however, most of the available scientific evidence comparing endovascular techniques in stroke does not take into account some of the empirical decisions made by the neurointerventionists behind the data. By not discriminating the data enough, we lose the ability to analyze these decisions, whether right or wrong. It could be the case that anterior circulation occlusions behave differently according to the specific site of occlusion and that, for instance, the anatomical differences between M1 and intracranial internal carotid artery (IC-ICA) occlusions render them different mechanical properties, and hence different technical demands in thrombectomy. On the other hand, a lot of the large-scale analysis have been performed on data from trials that took place years ago, and the neurointerventional community has upgraded both their toolkit and their skillset considerably since then.

While the use of a ballon guide catheter (BGC) has been regarded as a way to obtain better recanalization rates [2–4], with a possible benefit even regardless of the primary technique used [5], the superiority of the combined technique over direct aspiration or stent-retriever alone is more controversial. There are several variations in the very way the combined technique is performed, from the relationship between the stent and the aspiration catheter and their retrieval, to the precise timing of aspiration—they constitute, as a whole, another limiting factor in the design and comparison of studies. The ASTER‑2 trial [6] and a recently published retrospective study [7] did not find significant differences in terms of outcome between the combined technique and stent-retriever alone in anterior circulation occlusions; however, subanalysis focusing on occlusion site are not available as of yet for either study.

Among the possible factors that weigh in on the decisions that can determine different approaches between professionals, is the site of occlusion. IC-ICA occlusions are associated with a worse outcome [8], and evidence in the scientific literature is, again, controversial. Studies that looked at IC-ICA occlusions, often limited by their relatively small samples [8–11], range from suggesting superiority of the aspiration technique versus stent retriever in the absence of BGC [9] or higher reperfusion rates if an aspiration attempt was followed by a stent retriever, rather than the contrary [12], to better reperfusion rates with the combined technique [10, 11].

The approach to IC-ICA occlusions has also been heterogeneous in the neurointerventional department of the Niguarda Ca’ Granda Hospital (NCGH), Milan, with the two main strategies since 2019 being 1) a direct aspiration first pass technique (ADAPT); and 2) the stent-retriever assisted vacuum-locked extraction (SAVE). This prompted a formal analysis of the department’s database which became the grounds for the study hereby presented.

The aim of this study was to understand if either endovascular technique was associated with better recanalization results in IC-ICA occlusions specifically; we compared the SAVE technique (with and without BGC—BGC-SAVE and noBGC-SAVE) with the ADAPT technique (without BGC) in IC-ICA occlusions.

## Methods

### Population

An observational and retrospective analysis was done based on the prospectively kept internal database of the Neuroradiology Department of NCGH in Milan. The sample was created by searching the database for all patients with IC-ICA occlusions from 1 January 2019 to 31 March 2021, and then excluding those with tandem occlusions or with presumed spontaneous dissections. The time window chosen for this study was based on the consideration that the endovascular technique could be considered up to date, after both BGC and the SAVE technique had become a routine practice in NCGH.

The ethics committee has been made aware of this study. Considering that the data was reviewed retrospectively and anonymously, according to Italian regulations, patient consent was not needed.

### Data

In each case, the following variables were collected:

- Age and gender
- Use of BGC
- First technique chosen (ADAPT vs. SAVE) and rescue maneuver (change in technique when the first one was unsuccessful)
- Distal embolization in the territory of the initially occluded vessel (DE) or to other territories (EOT), including embolization to the anterior cerebral artery, when previously shown patent in CT angiography
- Number of thrombectomy attempts (passes)
- Modified treatment in cerebral ischemia (mTICI) score (dichotomized as good if ≥ 2b and bad if < 2b)
- Administration of intravenous fibrinolytic agent
- Groin-to-recanalization time
- Deployment of stent (intracranial or extracranial)
- Nonocclusive carotid bulb stenosis

### Endovascular Technique

In line with the recommendations of the American Stroke Association and the European Stroke Organization [13], whenever eligible, patients presenting with acute IC-ICA occlusions within the last 4.5 h were first given IV thrombolysis and then quickly transferred to the angio suite for endovascular revascularization.

All endovascular procedures were performed through a transfemoral approach using an 8F short sheath introducer. The choice between conscious sedation and general anesthesia was taken on the basis of clinical characteristics of the patient, and mainly set by the anesthesiologist.

A long sheath 6F (Neuronmax by Penumbra Inc, Alameda, CA, USA; Cerebase by Cerenovus, New Brunswick, NJ, USA; InfinityPlus by Stryker, Kalamazoo, MI, USA; or Ballast by Balt, Montmorency, France) or a BGC (FlowGate 2 by Stryker) was placed in the cervical ICA as high as possible. Decisions regarding the guiding catheter used and the thrombectomy techniques employed were up to the operator’s subjective choice.

### ADAPT Technique

The largest distal access aspiration catheter available (0.068–0.074 inch) is advanced through a long sheath, over a 0.021–0.027 inch inner lumen microcatheter with a 0.014–0.018 inch microwire inside, until the site of the occlusion. The microcatheter and microguidewire are then removed, and the aspiration catheter is gently advanced while connected to the pump; aspiration is then turned on just before the aspiration catheter contacts the clot. After two to three minutes, the aspiration catheter is gently retracted into the long sheath, which is also coupled with manual aspiration.

### SAVE Technique

A BGC or a long sheath is used and the largest aspiration catheter available (0.068–0.074 inch) is advanced in the guiding catheter over a 0.021–0.027 inch inner lumen microcatheter, with a 0.014–0.018 inch microguidewire inside. The microcatheter is then advanced past the occlusion over the microguidewire to allow the deployment of a stent-retriever over the occlusion. The large-bore distal access catheter is then advanced to contact the proximal edge of the clot, under pump aspiration, in a similar fashion as described for the ADAPT technique, but in this case also engulfing the initial part of the stent. After a couple of minutes, the stent-retriever and the large-bore distal access catheter are pulled out from the guiding catheter as a unit, keeping the relative position they have with each other, under manual aspiration from the guiding catheter. If a BGC is being used, the balloon is inflated just before the traction on the stent-retriever.

If the first attempt did not achieve proper recanalization or if there was recanalization with DE or EOT, the operator was free to change the technique whenever it was considered adequate.

### Statistical Analysis

The R Project for Statistical computing from the R Foundation (version 4.1.1, Vienna, Austria) was used for the statistical analysis of this study. To check whether the continuous variables were normally distributed, a Shapiro-Wilk test was used. Considering that the distribution was never normal, the different groups were compared using the Wilcoxon test.

Fisher’s exact test was used for discrete variables; the odds ratio (OD) with 95% confidence interval (CI) was reported only when a significant difference was discovered.

The statistical analysis of the variables was then performed in two different ways:

1. Comparing ADAPT group with the noBGC-SAVE group
2. Dichotomizing the SAVE group according to which guiding catheter was used (BGC-SAVE vs. noBGC-SAVE)

## Results

Between 1 January 2019 and 31 March 2021, in the context of acute ischemic stroke, a total of 673 endovascular procedures were performed at our center. Among these, 91 were IC-ICA occlusions that were included in this study, 44 (48%) patients were female and the median age was 79 years. Intravenous fibrinolytic was administered to 32 (35%) patients and 89 (98%) had a good mTICI score. These variables were not statistically different between the subgroups in any of the statistical analysis performed (Table 1), particularly the achievement of a good mTICI score. A stent was deployed in 9 cases (6 intracranial and 3 extracranial) and 4 patients had a nonocclusive stenosis of the carotid bulb on the same side of the IC-ICA occlusion.

**Table 1:** Patient demographics considering the whole population and according to subgroups

Variables	All patients (*N* = 91)	ADAPT (*N* = 20)	BGC-SAVE (*N* = 32)	noBGC-SAVE (*N* = 39)	*P* value
Age (years)	79 (64–84)	78.5 (69.75–84.75)	75.5 (64.75–81)	80 (63.5–84)	0.5
Gender, female	44 (48%)	8 (40%)	16 (50%)	20 (51%)	0.7
IVT	32 (35%)	7 (35%)	11 (34%)	14 (36%)	1
mTICI, 2b or better	89 (98%)	20 (100%)	32 (100%)	37 (95%)	0.5

Age is displayed as median and IQR

*ADAPT* a direct aspiration first pass technique, *BGC-SAVE* balloon guide catheter and stent-retriever assisted vacuum-locked extraction, *noBGC-SAVE* stent-retriever assisted vacuum-locked extraction without a balloon guide catheter, *IVT* intravenous therapy, *mTICI* modified treatment in cerebral ischemia score

Tables 2 and 3 summarize the remaining variables and the statistical analyses. The ADAPT technique was the first choice in 20 (22%) patients and the SAVE technique in 71 (78%). A BGC was used in 32 (35%) of the total number of patients, all with the SAVE technique (45% of SAVE patients). There was never the need to replace the initially chosen guiding catheter (whether BGC or long sheath), as it was always possible to go up with the chosen catheter as high as deemed necessary.

**Table 2:** Comparison of variables when the sample is dichotomized according to which technique was first used

Variables	ADAPT (*N* = 20)	noBGC-SAVE (*N* = 39)	*P* value
EOT	3 (15%)	6 (15%)	1
DE	15 (75%)	17 (44%)	0.03
FPE	5 (25%)	20 (51%)	0.09
Number of passes	3	1	< 0.01
Groin-to-recanalization time^a^	56 (IQR 34.75–91.50)	35.5 (IQR 27–54.75)	0.04

*ADAPT* a direct aspiration first pass technique, *noBGC-SAVE* stent-retriever assisted vacuum-locked extraction without a balloon guide catheter, *EOT* embolization to other territories, *DE* distal embolization (in same territory), *FPE* first pass effect

^a^Minutes, displayed as medians and IQR (Q1, Q3)

**Table 3:** Comparison of variables by dichotomizing the SAVE technique group according to whether a BGC was used or not

Variables	BGC-SAVE (*N* = 32)	NoBGC-SAVE (*N* = 39)	*P* value
EOT	3 (9%)	6 (15%)	0.5
DE	10 (31%)	17 (44%)	0.3
FPE	20 (63%)	20 (51%)	0.5
Number of passes	1	1	0.8
Groin-to-recanalization time^a^	36.5 (IQR 25.5–47.25)	35.5 (IQR 27–54.75)	0.5

*BGC-SAVE* balloon guide catheter and stent-retriever assisted vacuum-locked extraction, *noBGC-SAVE* stent-retriever assisted vacuum-locked extraction without a balloon guide catheter, *EOT* embolization to other territories, *DE* distal embolization (in same territory), FPE first pass effect

^a^Minutes, displayed as medians and IQR (Q1, Q3)

**Fig. 1:**
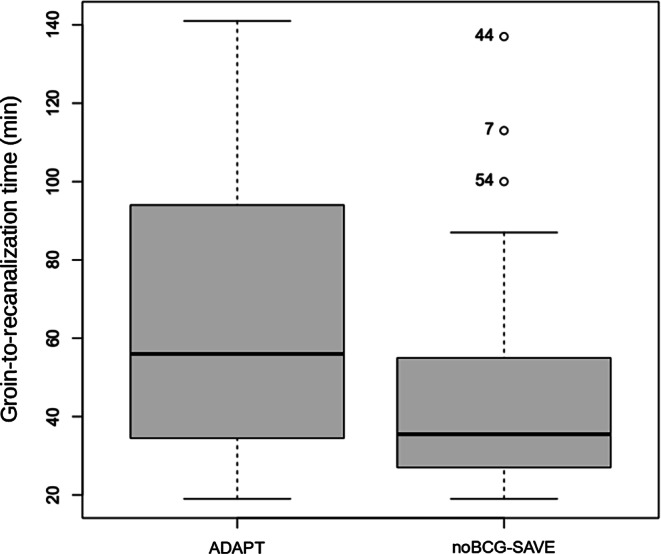
Boxplots of the groin-to-recanalization times comparing the ADAPT and the noBGC-SAVE groups. *ADAPT* a direct aspiration first pass technique, *noBGC-SAVE* stent-retriever assisted vacuum-locked extraction without a balloon guide catheter

There was DE in 15 (75%) of the patients when the ADAPT was the first choice, while in the noBGC-SAVE group, this happened in 17 (44%) cases (*p* = 0.03; OR 0.26, 95% CI 0.06–0.96). A FPE was possible in 5 (25%) patients in the ADAPT group and in 20 (51%) of the noBGC-SAVE group (*p* = 0.09); the median number of passes was, respectively, 3 and 1 (*p* < 0.01). The groin-to-recanalization (Fig. 1) was 56 (IQR 56.75) minutes in the ADAPT group and 35.5 (IQR 27.75) minutes in the SAVE group (*p* = 0.04). In the ADAPT group, a rescue maneuver with stent retriever was used in 16 (80%) cases, while a change in technique happened in 4 (10%) of the cases in which SAVE was the initial technique (*p* < 0.01; OR 0.03, 95% CI 0.005–0.154). Among the reasons for shift from ADAPT to SAVE technique, was the inability of the aspiration catheter to go up to the site of occlusion, namely due to difficulty progressing past the origin of the ophthalmic artery—this happened in 3 patients (15%).

**Fig. 2:**
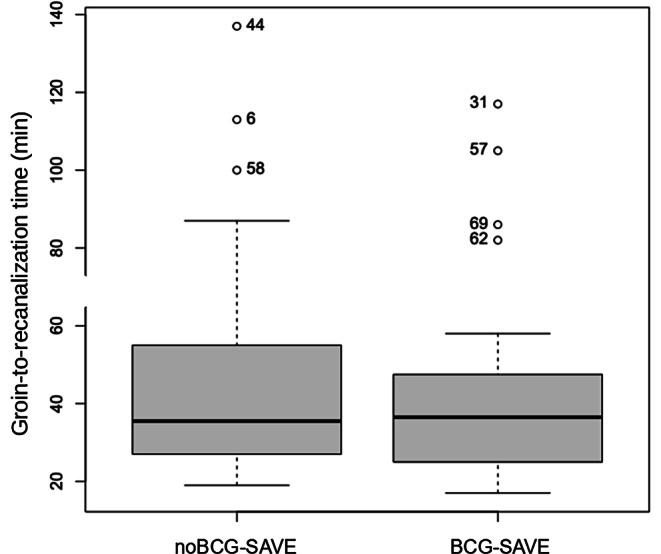
Boxplots of the groin-to-recanalization times comparing the noBGC-SAVE and the BGC-SAVE groups. *BGC-SAVE* balloon guide catheter and stent-retriever assisted vacuum-locked extraction, *noBGC-SAVE* stent-retriever assisted vacuum-locked extraction without a balloon guide catheter

In the SAVE technique group, DE occurred in 10 (31%) of the patients in which a BGC was used, versus 17 (44%) of those in which it was not (*p* = 0.3). Complete recanalization with a single pass (first pass effect—FPE) was achieved in 20 (63%) of the patients in the BGC group, and in 20 (51%) on the No-BGC group (*p* = 0.5). The median number of passes was 1 in both groups (*p* = 0.8). The groin-to-recanalization time (Fig. 2) was 36.5 (IQR 21.75) minutes in the BGC-SAVE group and 35.5 (IQR 27.75) minutes in the noBGC-SAVE group (*p* = 0.5).

The total number of EOT was 12 and there was no significant trend in any of the subgroup analyses: 3 (9%) and 3 (15%) in the BGC-SAVE and noBGC-SAVE groups (*p* = 0.5) respectively, and 6 (15%) in the ADAPT group (*p* = 1).

## Discussion

Different sites of occlusion might have different technical particularities and grouping them together on the basis of belonging to the same circulation might keep us from seeing these very differences, and understanding what the best approach is for each specific occlusion. The best technique for an M1 occlusion might not be the same for a carotid tip occlusion; however, even when looking at IC-ICA occlusions specifically, studies have been controversial [9–12]. The variety of tools at our disposal and differences in their very management might be in part responsible for keeping this an unsolved dilemma. This study tried to compare the two major technical decisions that take place in the endovascular treatment of IC-ICA occlusions.

In our sample, the combined technique without BGC, compared to the ADAPT, was associated with a lower rate of DE (44% vs. 75%, *p* = 0.03), a lower number of passes (1 vs. 3, *p* < 0.01), double the chances of FPE (51% vs. 25%, *p* = 0.09) and 20 min less of median procedure time (groin-to-recanalization: 35.5 min vs. 56 min, *p* = 0.03). The difference in mean number of passes was considered the most likely explanation for the faster median procedure times in the noBGC-SAVE group. In turn, the higher number of passes and the lower FPE in the ADAPT group might be due to its higher rate of DE.

Comparing the results obtained with and without BGC when the SAVE technique was used (BGC-SAVE vs. NoBGC-SAVE groups), despite a slight tendency for better results in the BGC-SAVE group, none of the variables reached statistical significance. In a study by Bourcier et al. [14], when comparing the BGC use with the combined technique without BGC in middle cerebral artery (M1 and M2) and ICA occlusions, there was no significant difference in the primary endpoint, which was obtaining nearly complete or complete recanalization (mTICI 2c/3). These findings coincide with ours, even though our recanalization rates were not set for nearly complete or complete recanalization.

The results of this study are also in line with others, namely the retrospective review done by Brehm et al. [10], in which the combined technique proved superior to the ADAPT in carotid T occlusions (*N* = 55), in both obtaining successful reperfusion (mTICI ≥ 2b) or near-perfect reperfusion (mTICI ≥ 2c). The rate of rescue maneuver in the ADAPT group was higher in our study (77%) than what is reported in the literature concerning anterior circulation occlusions altogether (around 30%) [10, 11, 14]; given the overall similar rates of successful recanalization, this might just reflect intricate differences in the resources with which one chases the goal of complete recanalization. Not much data are available in the literature concerning the rescue rates of ICA occlusions specifically. Brehm et al. noted that while the use of a combined technique might cost more than the ADAPT, this difference is offset by the more frequent need to resort to a rescue maneuver with stent retriever; however, such considerations vary greatly with the socioeconomic and political context of each hospital and between countries and were not the focus of this study.

Schönfeld et al. [4], by submitting patients to a follow-up MRI with DWI within 24 h of thrombectomy, showed that the benefit of BGC could go above and beyond what can be ascertained with the mTICI scale—such could also be the case with the combined technique when compared to aspiration, and it remains a concept to be proven.

While optimal, when possible the use of BGC could be limited by its stiffer properties when compared to other more flexible guide-catheters, and this poses a challenge in patients with long and tortuous vessels, namely in older patients. Although standard use of a BGC could be ideal whenever possible, our results suggest that the game-changing attitude to obtain better recanalization rates might be to use the SAVE technique as first line of treatment, rather than aspiration alone. Better results have been correlated with increasing sizes of aspiration catheters [15, 16], and even though in this study the aspiration catheters used were the largest that were commercially available, it may be the case that in ICA occlusions, the catheter-to-vessel ratio is not optimized. Along with a higher clot burden and the anatomical features of the ICA bifurcation, such could be the reasoning for considering the SAVE technique to be superior to aspiration alone in this specific site of occlusion.

Our findings contrast with the ones from Xing et al. [9], who reviewed a total of 109 terminal ICA occlusions and found that the recanalization rates (mTICI ≥ 2c) were higher (86.7% vs. 50%, *p* = 0.06) and the median puncture-to-reperfusion time was lower (38 minutes vs. 69 minutes, *p* = 0.001) in the aspiration group, compared with the stent-retriever group. The intermediate and aspiration catheters used in said study varied from 0.064 to 0.072 inches. The stent-retriever group also made use of aspiration, although only upon stent retrieval, and the SAVE technique, to our understanding, was not universally employed.

Lastly, the ASTER‑2 trial [6] and a recent study by Mohammaden et al. [7] did not find any significant differences in outcome between the combined technique and stent-retriever alone in anterior circulation occlusions. A subgroup analysis based on occlusion site could provide further relevant data.

The retrospective and observational nature of our study and its small sample size are important limitations, even though it is on par with the largest in the literature when it comes to recent studies that looked at ICA occlusions specifically [8–11]. In our study, the time frame was limited so that both the use of BGC and the up to date SAVE technique could be analyzed. The standard approach to ICA occlusions also differed between neurointerventionists, and this might have introduced a bias in the study. The experience each individual neurointerventionist and each department has with each technique (both ADAPT and SAVE) is also a factor to be considered, and could even explain differences between studies. We did not include the clinical outcome in this study to avoid biases in the evaluation of the success of the thrombectomy. We consider surrogates such as number of passes, procedure time, DE and FPE more faithful in representing the efficacy of each technique, as many other factors that come into play in the long-term outcome of patients may otherwise translate into equivocal interpretations in a sample of this size [17–19]. Finally, despite our results, a definite causal relation between location and better results depending on thrombectomy technique requires further studies. It also remains to be proven if other types of differences may exist in other sites of occlusion.

## Conclusion

In our study, the SAVE technique (stent-retriever assisted vacuum-locked extraction), compared with the ADAPT (a direct aspiration first pass technique), was associated with less distal embolization, more first pass effect, less passes and shorter procedural times, and these differences were greater than the ones seen when comparing the use of a balloon guide catheter with the SAVE technique. Such considerations warrant larger and, ideally, prospective studies.

## Abbreviations

ADAPT: A direct aspiration first pass technique
BGC: Balloon guide catheter
DE: Distal embolization to the same territory initially occluded
EOT: Embolization to other territories
FPE: First pass effect
IC-ICA: Intracranial Internal carotid artery
IQR: Interquartile range
mTICI: Modified treatment in cerebral ischemia
NCGH: Niguarda Ca’ Granda Hospital
SAVE: Stent-retriever assisted vacuum-locked extraction
